# Surgery in head and neck cancer: United Kingdom National Multidisciplinary Guidelines

**DOI:** 10.1017/S0022215116000475

**Published:** 2016-05

**Authors:** J J Homer

**Affiliations:** 1 Manchester Royal Infirmary, Manchester, UK; 2 Otolaryngology – Head and Neck Surgery, Manchester Academic Health Sciences Centre, University of Manchester, Manchester, UK

## Abstract

This is the official guideline endorsed by the specialty associations involved in the care of head and neck cancer patients in the UK. Surgery is one of the key modalities used in head and neck cancer treatment. Recent advances and a greater awareness of the short- and long-term toxicities associated with non-surgical modalities and newer technologies that permit minimal access resections have led to a resurgence in surgery. This paper provides an overview of the role of surgery in head and neck cancer practice.

The aim of surgery with curative intent in head and neck cancer (HNC) is complete microscopic surgical excision. Excision margins are a consistent prognostic factor^1^^–^^3^ and a major consideration for more radical post-operative adjuvant therapy (and therefore more attendant morbidity),^4^ with the possible exception of thyroid cancer.^5^ Whilst there has been considerable progress with less invasive surgical access techniques, the underlying principle of profound importance in head and neck surgery is that surgical resection achieves complete, microscopic clearance of the tumour with the appropriate safely margin according to the type, site and stage of cancer. There is virtually no oncological role for debulking surgery in order to improve the chances of cure with subsequent chemoradiation. Debulking may be necessary for airway preservation and for symptom palliation, however.

One of the most prominent surgical advances of recent times has been the development and popularisation of transoral access techniques for oropharyngeal, supraglottic and glottic cancers, via transoral laser microsurgery (TLM) and transoral robotic surgery (TORS). Transoral robotic surgery should be seen as an evolutionary refinement of TLM, especially useful for tongue base and supraglottic resections, and the evidence for these procedures should be considered together. When minimal access surgery is compared to open techniques, the advantages relating to reduction in morbidity are obvious. This applies to endoscopic approaches for sinonasal tumour resection, either with or without craniotomy. Here, the relevant comparison is to open transfacial access techniques, and the advantages of less radical access are obvious, with no compromise in prognosis (at least in selected cases).^6^ Any surgeon managing sinonasal tumours should be able to offer the full range of surgical techniques, open and endoscopic, and, as an oncology surgeon, be a core member of the multidisciplinary team.

However, with the transoral techniques of TLM and TORS, the relevant comparison is really to primary radiotherapy (RT) or chemoradiotherapy in the main. Even in glottic cancer, it has only been shown that there is equipoise between TLM and RT for T1a.^7^. There is less robust evidence for T1b cancers^8^ and clearly insufficient data for T2 glottic cancers and for supraglottic cancers. For oropharyngeal cancers, there is much work to do in order to define the role of transoral surgery in place of, or in concert with, chemoradiation.^9^ Much of this depends on whether and how post-operative RT and chemoradiotherapy can be modified in patients treated with primary surgery, and, especially for oropharyngeal cancer, the influence of human papilloma virus status and neck metastases. There appears to be consistent evidence that swallowing outcomes may be better in patients treated primarily with surgery, if post-operative treatment can be restricted to RT only and perhaps to a lower dose.^10^^–^^12^

A further issue with transoral techniques in particular is the proof of surgical margins. The practice of basing this on further biopsies or submission of additional tissue from the tumour bed has been shown to be less reliable in glossectomy and less prognostic than defining surgical margins from the main tumour.^13^ However, with small tumours, especially from the glottis, then: (a) smaller margins may be oncologically safe; and (b) the impact of thermal artefact is such that it is difficult to prove histological clearance without submitting separate material from the margins.^3^ The same issues apply to complex resections in which it can be very difficult for the pathologist to determine where the true margins are, for example with anterior and lateral skull base resections. The key is to good interdisciplinary working between surgeon and pathologist. The bottom line is that the determination of accurate surgical margins is critical, whatever the surgical technique employed.

For advanced disease, in which more radical, open surgery is required, the issues to consider are:

• Can a complete resection be achieved? If this is not realistic, then the morbidity of such surgery can rarely be justified
• Even if complete resection can be achieved, is the mortality risk and morbidity justified by the chances of overall survival?
• If radical surgery is to be done, it should be done comprehensively. There should be no compromise in the extent of the resection, when the attendant morbidity is not materially affected by a more radical approach with appropriate reconstruction in expert hands. This may mean pharyngolaryngectomy instead of laryngectomy, mandibulectomy instead of soft tissue resection only in the oral cavity or extending a maxillectomy posteriorly or superiorly.

For defects that will require reconstruction with microvascular free flaps, in most cases having two consultant surgeons has obvious advantages, regardless of specialties involved. The use of free flaps is increasing.^14^ There has been continued evolution of reconstruction options, with a greater variety of composite flaps suited to the defect involved. With regard to soft tissue reconstruction, the anterolateral thigh flap is ideal for most soft tissue defects,^15^ except when a thin flap might be required for smaller oral cavity defects.

When applying these principles to salvage surgery, these principles are even more important. The focus is defining what the role of salvage surgery is (cure, palliation) and what the chance of achieving the aim actually is in the setting of greatly increased chances of serious post-operative morbidity, with, in many cases, low chance of cure.^16^^–^^19^

With regard to neck dissection, for many N+ cases, conservation techniques allow the preservation of key non-lymphatic structures and the restriction of levels dissected according to the primary tumour and the amount of disease. Shoulder and neck dysfunction has been correctly recognised as an important contributor to quality of life after treatment. For N0 cases, it is reasonably clear which cases require elective neck dissection, when surgery is the primary treatment modality. In practice, when this is the case, the nature of surgery is such that the addition of a selective neck dissection adds little to the overall surgery. When this is not the case, there is a role for sentinel node biopsy.^20^ For neck disease treated with RT or chemoradiotherapy, neck dissection is only required for residual disease shown on conventional or positron emission tomography–computed tomography imaging.^21^

## Training and manpower in head and neck surgery

The situation in the UK contrasts with many other countries, in that HNC surgery is divided between the two major specialties of otorhinolaryngology–head and neck surgery (ORL–HNS) and oral and maxillofacial surgery (OMFS), in a more equitable fashion than most other countries. Should there continue to be the distinction of, in general, OMFS managing and operating on oral cavity cancer and performing most microvascular reconstruction, with ORL–HNS managing the pharynx, larynx and thyroid? There are areas of overlap, but the division largely remains, irrespective of the influence of interface interdisciplinary fellowships.

There is no consensus about the volume of major surgery required in order to achieve and maintain competence. Whilst there is a clear relationship between both hospital and individual surgeon volume with better outcomes, it is difficult to define a minimal cut-off in terms of volumes required.^22^ Even with something as easily defined as microvascular free flap surgery (with easily measurable outcomes), it many come as a surprise that there is no guidance on how may free flaps a surgeon or hospital should do per year in order to maintain and evidence competence.

In summary, the evolution of surgery for HNC continues to give rise to the ability to perform more complex tumour ablation together with more refined reconstruction and, at the same time, there has been significant progress in minimal access techniques without oncological compromise. The increasing use of chemoradiotherapy means there is an increase in salvage surgery (when appropriate) which always represents the most difficult challenge for a head and neck surgeon. These changes make the need for the clarification of training and minimal volumes for surgeons and hospitals even more important.
